# Effect of a Carotenoid Extract from *Citrus reticulata* By-Products on the Immune-Oxidative Status of Broilers

**DOI:** 10.3390/antiox11010144

**Published:** 2022-01-10

**Authors:** Alexandros Mavrommatis, Maria-Eleftheria Zografaki, Sofia Marka, Eleni D. Myrtsi, Elisavet Giamouri, Christos Christodoulou, Epameinondas Evergetis, Vasilios Iliopoulos, Sofia D. Koulocheri, Georgia Moschopoulou, Panagiotis E. Simitzis, Athanasios C. Pappas, Emmanouil Flemetakis, Apostolis Koutinas, Serkos A. Haroutounian, Eleni Tsiplakou

**Affiliations:** 1 Laboratory of Nutritional Physiology and Feeding, Department of Animal Science, School of Animal Biosciences, Agricultural University of Athens, Iera Odos 75, GR-11855 Athens, Greece; mavrommatis@aua.gr (A.M.); elenamirtsi@aua.gr (E.D.M.); egiamouri@aua.gr (E.G.); c.christodoulou@aua.gr (C.C.); epaev@aua.gr (E.E.); heliopoylos@gmail.com (V.I.); skoul@aua.gr (S.D.K.); apappas@aua.gr (A.C.P.); sehar@aua.gr (S.A.H.); 2 Laboratory of Molecular Biology, Department of Biotechnology, School of Applied Biology and Biotechnology, Agricultural University of Athens, Iera Odos 75, GR-11855 Athens, Greece; marielazog@gmail.com (M.-E.Z.); sofiamarka94@gmail.com (S.M.); mflem@aua.gr (E.F.); 3 Laboratory of Cell Technology, Department of Biotechnology, School of Applied Biology and Biotechnology, Agricultural University of Athens, Iera Odos 75, GR-11855 Athens, Greece; geo_mos@aua.gr; 4 Laboratory of Animal Breeding & Husbandry, Department of Animal Science, Agricultural University of Athens, Iera Odos 75, GR-11855 Athens, Greece; pansimitzis@aua.gr; 5 Laboratory of Food Process Engineering, Department of Food Science and Human Nutrition, Agricultural University of Athens, Iera Odos 75, GR-11855 Athens, Greece; akoutinas@aua.gr

**Keywords:** liver, bursa of Fabricius, spleen, antioxidants, immune, meat, antimicrobials, Liquid Chromatography-Mass Spectrometry (LC/MS-MS)

## Abstract

Although carotenoids generally possess antimicrobial and antioxidant properties, the in vivo synergistic action of carotenoid blends derived from plant-based by-products has not been thoroughly studied. Therefore, the carotenoid characterization and antimicrobial potential of *Citrus reticulata* extract as well as the impact of this carotenoid-rich extract (CCE) dietary supplementation on the performance, meat quality, and immune-oxidative status of broiler chickens were determined. One hundred and twenty one-day-old hatched chicks (Ross 308) were allocated to two dietary groups, with four replicate pens of 15 birds each. Birds were fed either a basal diet (CON) or the basal diet supplemented with 0.1% CCE (25 mg carotenoid extract included in 1 g of soluble starch) for 42 d. β-Cryptoxanthin, β-Carotene, Zeaxanthin, and Lutein were the prevailing carotenoid compounds in the *Citrus reticulata* extract. The CCE feed additive exerted inhibitory properties against both Gram-positive (*Staphylococcus aureus*) and negative (*Klebsiella oxytoca, Escherichia coli,* and *Salmonella typhimurium*) bacteria. Both the broiler performance and meat quality did not substantially differ, while the breast muscle malondialdehyde (MDA) concentration tended to decrease (*p* = 0.070) in the CCE-fed broilers. The inclusion of CCE decreased the alanine aminotransferase and MDA concentration, and the activity of glutathione peroxidase, while the activity of superoxide dismutase was increased in the blood. Catalase and NADPH oxidase 2 relative transcript levels were significantly downregulated in the livers of the CCE-fed broilers. Additionally, Interleukin 1β and tumor necrosis factor (*TNF*) relative transcript levels were downregulated in the livers of the CCE- fed broilers, while *TNF* and interferon γ (*IFNG*) tended to decrease in the spleens and bursa of Fabricius, respectively. The present study provided new insights regarding the beneficial properties of carotenoids contained in *Citrus reticulata* in broilers’ immune-oxidative status. These promising outcomes could be the basis for further research under field conditions.

## 1. Introduction

Although antimicrobials experienced their golden era between the 1950s and 1970s, they were later characterized as one of the most important threats to humanity [1]. The bacteria selection mechanisms towards antibiotic resistance led to the ban of using antibiotics as antimicrobial growth promoters (AGP) in livestock, which led to the evaluation of alternative solutions [2]. Thenceforth, researchers are scavenging a natural toolbox of bioactive compounds contained in plants as alternatives to antibiotics to promote growth performance and enhance host health [3].

In this light, a wide variety of essential oils (EOs) derived from oregano (thymol, carvacrol), cinnamon (cinnamaldehyde), garlic, rosemary, citrus, etc. have been explored solely or as blends to improve animal health and performance [3]. Without narrowing out the imperative necessity for alternatives to antimicrobials, there is also a growing consumer demand for the replacement of synthetic with natural antioxidants since the use of synthetic molecules has been linked with possible toxicity on the liver and carcinogenesis in animal and human studies [4].

Bioactive compounds presented in *Citrus* (*Rutaceae* family) may be of high importance in animal nutrition since they exert both antimicrobial and antioxidants properties in vitro [5]. However, the competition amongst food and feed for resources increasingly suppresses the availability of various promising compounds rich in bioactive substances in animal nutrition.

Within the EU, more than 10 Mt of *Citrus* fruit are produced annually, and Spain, Italy, and Greece have the leading roles. However, the production of *Citrus* fruits far exceeds consumers’ demands for fresh consumption. A significant volume of this production is originated in the food industry and majorly for juice, jams, beverages, etc. [6]. Amongst these fruits, 50–60% are discarded, and consequently plenty of difficult to stock sought-after bioactive compounds are wasted as well [7].

By-products from the *Citrus* juice industry mainly consist of two fractions; peel and pulp. Both fractions are considered a great source of bioactive compounds, including dietary fiber, minerals, organic acids, vitamins, phenolic acids, flavonoids, terpenes, carotenoids, etc. [8]. Amongst the aforementioned, carotenoids have gained the attention of animal scientists due to their promising health-promoting properties, including immunomodulatory, anti-inflammatory, antibacterial, and antioxidant effects [8].

The poultry industry is considered one of the most challenging sectors of livestock when the expansion of animals’ health and products quality are concerned. Various synthetic substances and feed additives are used by poultry producers to enhance the productivity of their birds especially after the prohibition of AGP [9]. In this scenario, valorization of plant-based wastes and their bioactive compounds have gained popularity in the poultry sector aiming to improve production performance, birds’ health, and product quality [8,9].

Interestingly, the supplementation of laying hens’ diet with 8 mg/kg canthaxanthin improved egg performance while simultaneously boosting reproduction indices [10]. It was also observed that the supplementation of 2% and 4% of fresh lemon (rich in carotenoids) improved the production performance, immune status, and antioxidative status of laying hens during the late laying period [11]. Dietary supplementation with either 2.5% or 5% tomato powder (rich in carotenoids) improved the oxidative stability by reducing the malondialdehyde (MDA) concentration in the breast and liver of Japanese quail, while the pro-inflammatory indices, such as nuclear factor kappa beta were also downregulated in their livers [12,13].

Notably, the inclusion of 6 mg/kg canthaxanthin in broiler diets decreased the concentration of the thiobarbituric acid reactive substance (TBARS) [14] and increased the total antioxidant capacity in the liver of duck progeny [15]. Similarly, the inclusion of 75 mg/kg lycopene in broiler diets improved meat oxidative stability [16]. Furthermore, the supplementation with 5% lycopene increased the activity of the antioxidant enzyme in the blood of heat-stressed broilers [17], while the inclusion of 6 mg/kg canthaxanthin in duck progeny decreased the superoxide dismutase activity, MDA, and protein carbonyls concentration in the liver [15].

Although the antioxidant and immunomodulatory properties of individual carotenoid substances have been investigated in poultry, scarce information exists regarding the effect of natural derivatives obtained as agro-industrial by-products while no evidence exists about the effect of *Citrus reticulata* (mandarine) extract in broilers.

Considering the abovementioned issues, the preliminary scope of the present study was to characterize the carotenoids profiling of *Citrus reticulata* juice by-products and their potential as antimicrobial alternatives. Secondly, the principal aim of our study was the technological inclusion of the carotenoid extracts on diet and the impact on broilers’ performance and meat quality, antioxidant status, and transcriptional profiling of immune-related genes.

## 2. Materials and Methods

### 2.1. Solvents and Standards

Zeaxanthin (≥98%), lutein (≥95%), β-cryptoxanthin (≥97%) standards were purchased from ExtraSynthese (Genay, France), α-carotene (≥95%) standard was obtained from CaroteNature (Münsingen, Switzerland), astaxanthin, β-carotene, and fucoxanthin (≥95%) standards were provided from Sigma-Aldrich (St. Louis, MO, USA), and canthaxanthin (≥93%) was from Honeywell (Charlotte, NC, USA). LC-MS grade methanol solvent was obtained from JT Baker and absolute ethanol from Fisher Chemical (Chicago, IL, USA). The analytical grade solvents isopropanol, methanol, dichloromethane, and diethyl ether were purchased from Fisher Chemical and petroleum ether (40–60 °C), chloroform, and dimethyl sulfoxide from Carlo Erba (Val-de-Reuil, France), Fisher Chemical (Chicago, IL, USA), and Sigma-Aldrich (Chicago, IL, USA), respectively. Potassium hydroxide (KOH) was purchased from Honeywell (Charlotte, NC, USA).

### 2.2. Carotenoids Supplement

#### 2.2.1. Cold Pressure Essential Oil (CPEO) Extraction of *Citrus reticulata*

CPEO from *Citrus reticulata* industrial processing used as a raw material of the study was kindly provided by Christodoulou Bros SA (Argolis, Greece) fruit juices industry.

##### Separation of the CPEO Non-Volatile Fraction

The non-volatile fraction was separated from *Citrus reticulata* CPEO as azeotropic mixture with isopropanol, which was evaporated under reduced pressure at 30 °C temperature. The azeotropic mixture of isopropanol with CPEO’s volatile components was removed under these conditions, using a BÜCHI Rotary Evaporator apparatus (Rotavapor R-210, Vacuum Controller V-850, Heating Bath B-491, Vacuum Pump V-700, Flawil, Switzerland). All procedures were performed in darkness to avoid the degradation of the components.

##### Content of CPEO’s Non-Volatile Fraction

The non-volatile fraction of CPEO contained a variety of bioactive molecules, including carotenoids. In specific, the azeotropic removal of the main volume of volatiles, resulted in the sharp decrease of the prevailing volatile component D- limonene from 85% to 2%, as was determined by GC-MS analysis following the methodology reported by Kapsaski-Kanelli et al. [18] and the complete removal of the remaining volatiles, such as α-pinene, β-myrcene, and β-thujene. On the other hand, we detected traces of the less volatile molecules, such as citronellal, decanal, citronellol, copaene, δ-cadinene, α-sinensal, nerol acetate, D-germacrene, citronellol acetate, α-panasinsene, caryophyllene, intermedeol, carvone, hedycaryol, Z-2-dodecenol, humulene, α-farnesene, 2-caren-4-ol, terpinyl acetate, trans-limonene oxide, and cis-carveol.

In respect to the non-volatile fraction, the presence of the following flavonoids and coumarins was verified in respect to a previous report [19]: the polymethoxyflavones nobiletin, 5,6,7,4′-tetramethoxyflavone, 3,5,6,7,8,3′,4′-heptamethoxyflavone, and tangeretin, the three furanocoumarins heraclenol, 8-geranyloxypsoralen, and bergamottin and the coumarin, auraptene.

##### Preparation of *Citrus reticulata*-Based Feed Additive

The dried carotenoid extract from *Citrus reticulata* was used to prepare the administration formula. For this purpose, 10 g of carotenoid extract was diluted in 400 mL of water and then 400 g of starch (Starch soluble GR for analysis ISO. CAS 9005-84-9, Sigma-Aldrich, St. Louis, MO, USA). The solution was homogenized in an ultrasonic bath and then subjected to deep freezing (−80 °C). Finally, the frozen solution was dehydrated in a freeze dryer, concluding the administration formulation.

#### 2.2.2. Carotenoid Characterization

##### Saponification

After the removal of the volatile fraction and prior to chromatographic analysis the remaining residue of *Citrus reticulata* CPEO was saponified utilizing a modification of the method proposed by Hart & Scott [20]. More specifically, 100 mg of the residue were diluted in 2 mL of a petroleum ether/diethyl ether 70:30 mixture and an equal volume of 10% KOH in methanol was added. The mixture was stirred overnight in darkness at room temperature and then was poured into a centrifuge tube containing 2 mL water and centrifuged at 5000 rpm for 2 min. The aqueous-alcoholic phase was backwashed twice with 2 × 2 mL of petroleum ether and diethyl ether (1:1) mixture. All organic phases were combined, washed three times with 3 × 2 mL of water, and evaporated to dryness under reduced pressure to provide the saponified carotenoid extract [21].

##### Preparation of Standard Stock Solutions

For each analyte, a stock solution was prepared at concentrations ranging from 500 to 1200 μg/mL. Zeaxanthin, lutein, β-cryptoxanthin, α-carotene, and β-carotene were diluted in chloroform, astaxanthin, and canthaxanthin in dichloromethane and fucoxanthin in dimethyl sulfoxide. Immediately before each analysis, the stock solutions of analytes were diluted in methanol for the preparation of standard solutions containing the analytes at concentrations ranging from 50 to 2000 ng/mL. These solutions were utilized for the construction of the respective calibration curves. All stock solutions were maintained at −20 °C.

##### Liquid Chromatography-Mass Spectrometry (LC/MS-MS)

The identification of carotenoid components was performed using an Accela Ultra High-Performance Liquid Chromatography system as described by [4].

The analyte separation was performed on a C30 column of internal diameter 3 × 150 mm and particle size 2.7 μm (HALO C30, Advanced Materials Technology, Wilmington, NC, USA). Methanol was used as mobile phase A and ethanol as mobile phase B. The injection volume of each sample was 10 μL, and the tray and column temperatures were set at 10 and 12 °C, respectively. Gradient elution conditions for mobile phases A and B were set as follows: 0.0–20.0 min, from 0% to 40% B; 20.0–22.0 min, 40% B; 22.0–25.0 min from 40% to 60% B; 25.0–26.0 min, 60% B and 26.1–28.0 min 0% B for the re-equilibration of column between two injections. The flow rate was set at 0.5 mL/min.

For the MS/MS determination, the Atmospheric-Pressure Chemical Ionization (APCI) technique was utilized. Molecular ion transitions and collision energies of the analytes were determined by direct infusion in full scan in a mass range from 100 to 1400. The respective gas pressures were set at 30 and 5 Arb, respectively, the discharge current was set at 4.0 μA, the capillary and the vaporizer temperature was regulated at 270 °C and 450 °C, respectively, and the collision pressure of Argon gas was adjusted at 0.2 Pa.

The signals of the selected ion transitions of the deprotonated molecules of m/z which were used: Fucoxanthin (658.821 > 598.080 (21 eV)/657.970 (18 eV)) and lutein (568.682 > 535.810 (29 eV)/550.654 (21 eV)). The signals of the selected ion transitions of the protonated molecules of m/z were used are: Astaxanthin (597.199 > 125.263 (31 eV)/153.070 (23 eV)), zeaxanthin (569.219 > 95.568 (33 eV)/123.255 (34 eV)), canthaxanthin (564.547 > 157.233 (25 eV)/174.005 (27 eV)), β-cryptoxanthin (553.218 > 95.594 (48 eV)/138.223 (26 eV)), α-carotene (537.265 > 122.898 (30 eV)/413.359 (17 eV)), and β-carotene (537.246 > 118.844 (30 eV)/177.270 (19 eV)) (Table 1).

### 2.3. Antimicrobial Potential of Carotenoids Feed Additive

#### 2.3.1. Preparation of Extract

The CCE additive was extracted with hexane: ethanol: acetone (2:1:1) at a raw material to solvent ratio of 1:10 (*w*/*v*). Extraction was performed by Bead milling on the Bead Ruptor 4 (Omni International, Kennesaw, GA, USA) using 2.8 mm ceramic beads. After overnight maceration, the resulting extract was centrifuged at 16,000× *g* at 4 °C for 10 min and filtered using 0.22 μm syringe filter (Millipore PES Membrane, Macherey-Nagel GmbH & Co. KG, Düren, Germany). Afterward, the solvent was evaporated by use of concentrator plus vacuum rotator (Eppendorf, Hamburg, Germany), and then the dried extract was reconstituted with pure dimethylsulfoxide (DMSO) to various concentrations at a 5% final concentration of DMSO.

#### 2.3.2. Microbial Strains

The antimicrobial activity assay of the extract was investigated against three Gram-negative (*Escherichia coli, Klebsiella oxytoca,* and *Salmonella typhimurium*) and one Gram-positive (*Staphylococcus aureus* (ATCC 25923)) bacteria strains. *E. coli, Klebsiella oxytoca,* and *Salmonella tiphymurium* were obtained from the Laboratory of Cell Technology, Department of Biotechnology, Agricultural University of Athens. All tested strains were grown in Mueller–Hinton (MH) broth overnight under agitation at 37 °C. Afterward, the turbidity of test organisms was adjusted using 0.5 McFarland standard at a final concentration of 10^8^ CFU/mL [5].

#### 2.3.3. Measurement of Viable Bacteria Using MTT Viability Assay

Antibacterial effects of the extract on cell viability were assessed using MTT [(3-(4,5-dimethylthiazol-2-yl)-2,5-diphenyltetrazolium bromide] assay with some modifications [22]. Briefly, overnight cultures of tested bacteria were diluted 100-fold in MH broth, and aliquots (50 μL/well) of cell suspensions were transferred into a round-bottomed 96-well microplate. 

Different concentrations (50 μL) of CCE extract were added, and the microplate was incubated at 37 °C for 12 h. DMSO 5% concentration was used as a negative control. Then, 20 μL of MTT Reagent (5 mg/mL) was added to all wells, and the plate was incubated for 30 min on a thermostatic shaker at 37 °C and 200 rpm in the dark. DMSO (100 μL) was added followed by an incubation of cultures for 10 min at 37 °C. The absorbance of the reaction solution was measured at 560 nm using a microplate reader (Infinite M200 PRO, Tecan, Männedorf, Switzerland).

### 2.4. Broilers’ Trial

The experimental procedure of this study continued the analytical procedures initiated in our previous work [4]. One hundred and twenty as hatched (*n* = 120), 1-day-old, Aviagen Ross 308 broilers were allocated to two experimental treatments for 42 days. Each treatment had four floors of replicate cages of 15 broilers each. More information about the EU guidelines for animals used for scientific purposes, temperature, and lighting programs are available by Mavrommatis et al. [4].

#### 2.4.1. Diets’ Formulation

Broilers were fed three different diets depending on the growing phase (Table 2). In the control (CON) group, broilers were fed a basal diet, while in the CCE group, carotenoids derived from *Citrus reticulata* using soluble starch for its inclusion (25 mg of pure carotenoid extract per 1 g of starch) were added to the starter, grower, and finisher diet at a level of 0.1% (1 g/kg feed; Table 2). 

The inclusion level of the carotenoid extract was set taking into account the limits of EFSA on canthaxanthin [23] and the equivalents of *Citrus reticulata* carotenoids to retinol [24]. Feed and water were provided ad libitum. Experimental diets from the three growing phases were milled through a 1-mm screen before analysis. Diets chemical composition was performed as described by Mavrommatis et al. [4] and determined and calculated analyses are presented in Table 2.

#### 2.4.2. Determination of Performance Parameters

Bodyweight (BW) was recorded on the first day of the experimental period and at the end of each feeding phase. The feed intake was recorded and feed conversion ratio (FCR) was calculated. The total mortality was calculated as the number of broilers that died throughout the study compared to the initial number of broilers placed.

#### 2.4.3. Sample Collection

On day 42 of age, 16 broilers (8 per treatment and two per replicate pen) were randomly selected and were euthanized using an electrical stunning device prior to slaughter. Approximately, 6 mL of whole blood were immediately collected into both heparin-containing tubes (170 units heparin; BD Vacutainer, Plymouth, UK) and no-anticoagulant tubes. 

Then, the blood samples were centrifuged at 2500 rpm at 4 °C for 15 min to obtain plasma and/or serum from the cells. Liver, spleen, and bursa of Fabricius tissues were also carefully excised and immediately snap-frozen, and subsequently stored at −80 °C for further analyses. The carcasses after chilling at 4 °C for 24 h, were weighed to estimate the percentage of carcass yield while the right part of the breast muscle was removed from the cold carcass and used for the determination of meat quality indices (pH_24_, color, shear force, and cooking loss).

#### 2.4.4. Physical and Color Traits

The pH was measured via the insertion of an electrode attached to a pH meter (Sentron 1001 pH System, Roden, the Netherlands) 24 h post-mortem into the right section of the breast muscle. Two buffers and pH 4.0 and 7.0 at room temperature were used for the calibration (Merck, Darmstadt, Germany). Color traits were determined in the breast muscle after 30 min at room temperature, and for every sample, there were two measurements implemented. A Miniscan XE (HunterLab, Reston, VI, USA) was used to determine color using the Hunter Lab L* (lightness), a* (redness), b* (yellowness) system CIE [25], which was standardized using white and black tiles.

For the determination of cooking losses, the right section of breast muscle was weighed, placed in plastic bags, cooked for 30 min at 80 °C in a temperature-controlled water bath. Subsequently, samples were left under running tap water for 15 min, dried, and weighed to measure the percentage of cooking loss. For the evaluation of shear force, the method published by Cason et al. [26] was used. In detail, the shear force was measured using the Zwick Testing Machine (Model Z2.5/TN1S; Zwick GmbH & Co, Ulm, Germany) equipped with a shear blade (Warner-Bratzler G146; Instron, Grove City, PA, USA), and three strips from the breast muscle of 1 cm^2^ were cut parallel to muscle fibers. The peak force values were recorded in N/mm^2^.

#### 2.4.5. Molecular Analysis

##### RNA Isolation and cDNA Synthesis

The total RNA was isolated from the liver, spleen, and bursa of Fabricius tissue samples of broilers separately using Trizol (Invitrogen, Carlsbad, CA, USA) according to the manufacturer’s instructions. Pure RNA treatment with DNase, purification, and cDNA synthesis were described by Mavrommatis et al. [27].

##### Primers’ Design

A pair of primers specific for *GAPDH*, Glutathione Peroxidase 1 (*GPX1*), Glutathione Peroxidase 2 (*GPX2*), NADPH oxidase 1 (*NOX1*), NADPH oxidase 2 (*NOX2*), NADPH oxidase 3 (*NOX3*), Mitogen-activated protein kinase (*MAPK*), Catalase (*CAT*), Superoxide Dismutase 1 (*SOD1*), Glutathione Transferase A2 (*GSTA2*), Nitic Oxide Synthase 2 (*NOS2*), and Beta-actin (*ACTB*) Toll-like receptors 4 (*TLR4*), Nuclear factor-kappa B (*NFKB*), Tumor necrosis factor (*TNF*), Translation initiation factor IF-1 (*INFA*), Interferon-gamma (*INFG*), Interleukin 1 Beta (*IL1B*), Interleukin 2 (*IL2*), Interleukin 6 (*IL6*), C-X-C Motif Chemokine Ligand 8 (*IL8*), and Interleukin 18 (*IL18*) have been initiated by Mavrommatis et al. [4,27] (Table 3). The specificity of each pair of primers was tested through the dissociation curves, and the amplification products were subjected to agarose gel electrophoresis (2%) to confirm the production of a single amplicon per reaction.

##### Real-Time Quantitative PCR

The relative mRNA expression levels for the target genes were quantified with a StepOnePlusTM Real-Time PCR System (Applied Biosystems, Foster City, CA, USA) as described by Mavrommatis et al. [27] using *GAPDH* and *ACTB* as housekeeping genes to normalize the cDNA template concentrations [27,28].

#### 2.4.6. Biochemical Analyses

##### Hematological and Biochemical Parameters in Blood

Blood serum from 16 broilers at 42 d of age in total (eight per treatment and two per replicate pen) was collected as already stated above to examine selected haematological and biochemical parameters. In detail, aspartate aminotransferase (SGOT-AST) (IU/L), alanine aminotransferase (SGPT-ALT) (IU/L), blood urea nitrogen (BUN) (mg/dL), γ-glutamyltransferase (γ-GT) (IU/L), alkaline phosphatase (IU/L), cholesterol (mg/dL), total proteins (g/dL), and fractions of albumins (g/dL) and globulins (g/dL) were assessed using an automatic ABX Pentra 400 analyzer (Horiba-ABX, Montpellier, France).

##### Antioxidant Enzymes Activities and Oxidative Status Indicators in Blood Plasma

The assays for antioxidant enzyme activities, oxidative stress indicators, and the total antioxidant capacity were performed using a UV/Vis spectrophotometer (GENESYS 180, Thermo Fisher Scientific, Massachusetts, USA) as previously described [4,29].

##### Breast Muscle Antioxidant Status

Lipid peroxidation activity in breast muscles was assessed using the method of Park et al. [30] as previously described by Mavrommatis et al. [4], while breast sample were also extracted for the determination of total antioxidant capacity using a modified method by Martínez et al. [31] as previously described by Mavrommatis et al. [4].

##### Breast Muscle Fatty Acid Profile

Breast tissue samples were partially thawed at 4 °C and trimmed to remove any external adipose and connective tissue. The total fatty acids (FA) were extracted and methylated directly, according to the method of O’ Fallon et al. [32]. For the determination of FA profile, an Agilent 6890 N gas chromatograph equipped with an HP-88 capillary column (60 m × 0.25 mm i.d. with 0.20 μm film thickness, Agilent, Santa Clara, CA, USA) and a flame ionization detector (FID) was used. Each peak was identified and quantified using a 37 component FA methyl esters (FAME) mix standard (Supelco, Sigma-Aldrich, St. Louis, MO, USA).

### 2.5. Statistical Analysis

The dataset was evaluated in SPSS.IBM software (v 20.0), and the results are depicted as mean ± standard error of means (SEM). For broiler growth performance, each experimental unit consisted of the replicate pen, while for molecular and biochemical analyses, the experimental unit considered the animal. The data were analyzed using independent-samples *t*-test comparing CCE to the CON group. Simplifying the visualization of these results, GraphPad Prism 6.0 (2012) depicted interleaved bars ± SEM. Statistical significance was set at *p* < 0.05.

## 3. Results

### 3.1. Carotenoid’s Composition in Feed Additive

Amongst the fractions, β-Cryptoxanthin was the prevailing carotenoid, while β-Carotene, Zeaxanthin, and Lutein were followed (Table 4). In the final product, which was used as a feed additive, only traces of α-Carotene and Astaxanthin were determined, while Canthaxanthin and Fucoxanthin were not determined at all (Table 4).

### 3.2. In Vitro Antimicrobial Potential of Citrus reticulata Feed Additive

**Figure 1:**
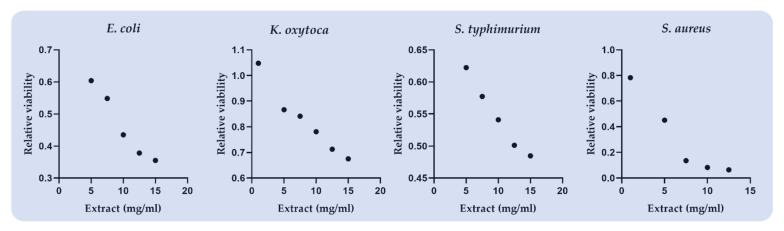
The effect of *Citrus reticulata* carotenoid extract included in soluble starch in the relative viability of *E. coli, K. oxytoca, S. typhimurium,* and *S. aureus* using MTT assay.

The effects of *Citrus reticulata* carotenoid extract on the relative bacterial viability are presented in Table 5 and Figure 1. The CCE extract depicted the maximum antimicrobial potential (IC50 = 4.22 mg/mL) against the Gram-positive strain *Staphylococcus aureus* (Table 5; Figure 1). Gram-negative strains demonstrated higher antimicrobial resistance against CCE extract compared to the Gram-positives. More specifically, *Escherichia coli, Salmonella typhimurium,* and *Klebsiella oxytoca* displayed a 50% inhibition in their relative viability using 8.63, 13.18, and 21.08 mg extract/mL, respectively (Table 5; Figure 1)

### 3.3. Broilers Measurement

#### 3.3.1. Growth Performance

The effects of feeding *Citrus reticulata* carotenoid extract on broiler body weight, feed intake, feed conversion ratio, carcass yield, liver, spleen, and bursa of Fabricius relative weight are presented in Table 6. Considering the whole experimental period (42 days), the growth performance was not considerably affected. Nevertheless, the carcass yield in CCE- fed broilers tended to increase (*p* = 0.093).

#### 3.3.2. Breast Meat Traits and Fatty Acid Profile

The effects of feeding *Citrus reticulata* carotenoid extract on broilers’ meat color and physical traits are presented in Table 7. The carotenoid supplementation did not affect the carcass color, and no major differences in physical traits were exhibited. More specifically, the cooking loss was significantly increased (*p* = 0.044) in the CCE-fed broilers (Table 7).

The effects of feeding *Citrus reticulata* carotenoid extract on broilers’ breast meat fatty acid profile are presented in Table S1. The carotenoids supplementation did not affect the breast meat fatty acid profile. However, the proportion of polyunsaturated fatty acid (PUFA) tended to increase (*p* = 0.096) in the breast meat of the CCE-fed broilers (Table S1).

#### 3.3.3. Breast Muscle Total Antioxidant Capacity and Lipid Peroxidation

**Figure 2:**
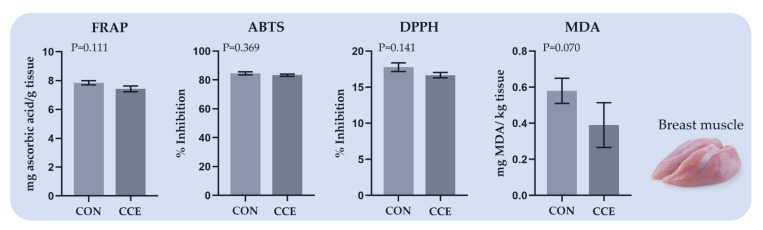
The total antioxidant capacity and lipid peroxidation index of breast muscle of broilers fed the two diets (Control; CON and carotenoid extract from *Citrus reticulata* included in soluble starch; CCE) at 42 days. *FRAP: Ferric Reducing Ability of Plasma, ABTS: 2,2′-azinobis-(3-ethylbenzothiazoline-6-sulfonate), DPPH: 2,2′-diphenyl-1-picrylhydrazyl radical,* *and MDA: Malondialdehyde*.

The effects of feeding *Citrus reticulata* carotenoid extract on the total antioxidant capacity and lipid peroxidation indicator of breast muscle of broilers are depicted in Figure 2. The total antioxidant capacity did not considerably differ, while the MDA concentration of breast muscle at 48 h at 4 °C tended to decrease (*p* = 0.070) in the CCE-fed broilers (Figure 2).

### 3.4. Haematological and Biochemical Parameters in Blood

**Figure 3:**
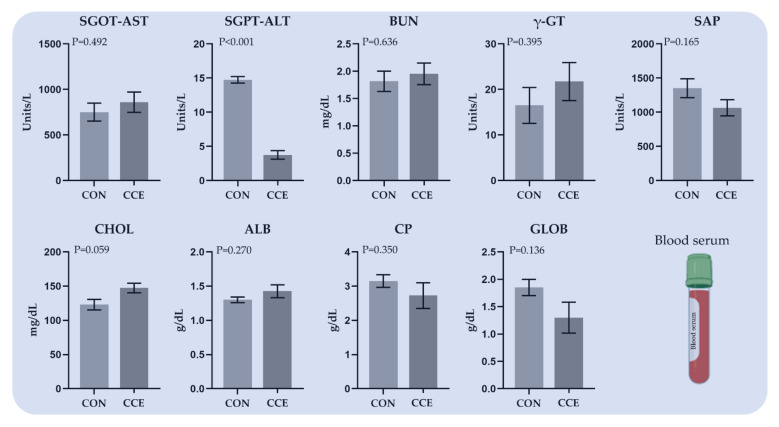
The mean and standard error of means (SEM) of selective hematological and biochemical parameters in blood serum of broilers fed the two experimental diets (Control; CON and carotenoid extract from *Citrus reticulata* included in soluble starch; CCE) at 42 days. *SGOT-AST: aspartate aminotransferase, SGPT-ALT: alanine aminotransferase, BUN: blood urea nitrogen, γ-GT: γ-glutamyltransferase, SAP: alkaline phosphatase, CHOL: cholesterol, and CP: total proteins and fractions of albumins (ALB) and globulins (GLOB)*.

The effects of feeding *Citrus reticulata* carotenoid extract on selective hematological and biochemical parameters in blood serum are presented in Figure 3. Despite a few numerical fluctuations, the investigated parameters were not considerably affected by the dietary treatments. Nevertheless, the activity of alanine aminotransferase (SGPT-ALT) was significantly decreased (*p* < 0.001) in the blood serum of CCE-fed broilers (Figure 3). On the other hand, the concentration of cholesterol (CHOL) tended to increase (*p* = 0.059) in the CCE-fed broilers’ serum (Figure 3).

### 3.5. Antioxidant Status

#### 3.5.1. Relative Transcript Levels of Genes Involved in Oxidative Status in the Liver

**Figure 4:**
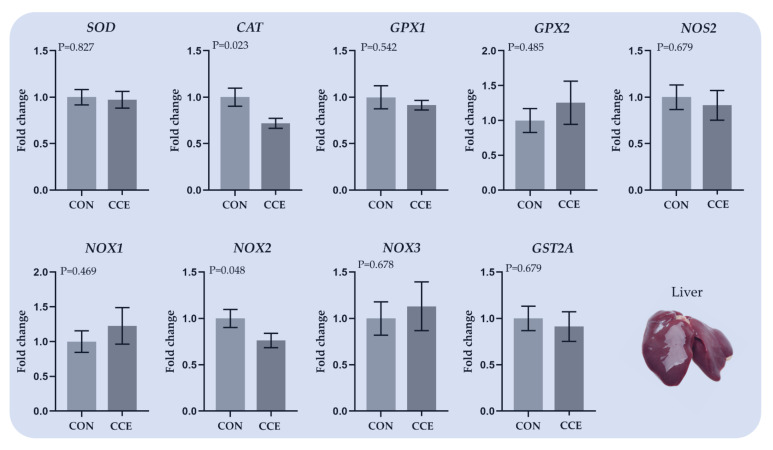
The mean and standard error of means (SEM) of relative transcript levels as fold changes of several genes involved in the antioxidant system in the liver of broilers fed the two experimental diets (Control; CON and carotenoid extract from *Citrus reticulata* included in soluble starch; CCE) at 42 days. *SOD: Superoxide Dismutase, CAT: Catalase, GPX1: Glutathione Peroxidase 1, GPX2: Glutathione Peroxidase 2, NOS2: Nitic Oxide Synthase 2, NOX1: NADPH oxidase 1, NOX2: NADPH oxidase 2, NOX3: NADPH oxidase 3, and GSTA2: Glutathione Transferase A2*.

The effects of feeding *Citrus reticulata* carotenoid extract on the relative expression of genes involved in oxidative mechanisms in the liver of broilers are presented in Figure 4. Although we did not observe a holistic alteration in the profiling of the investigated genes between CON and the CCE treatment, two interesting fluctuations were reported. More specifically, the relative transcript levels of *CAT* were significantly downregulated (*p* = 0.023) in the CCE-fed broilers’ liver (Figure 4). Moreover, the *NOX2* relative transcript levels were significantly decreased (*p* = 0.048) in broiler livers where carotenoid extracts were supplemented (CCE).

#### 3.5.2. Antioxidant Enzymes Activities, Total Antioxidant Capacity, and Oxidative Status in Blood Plasma

**Figure 5:**
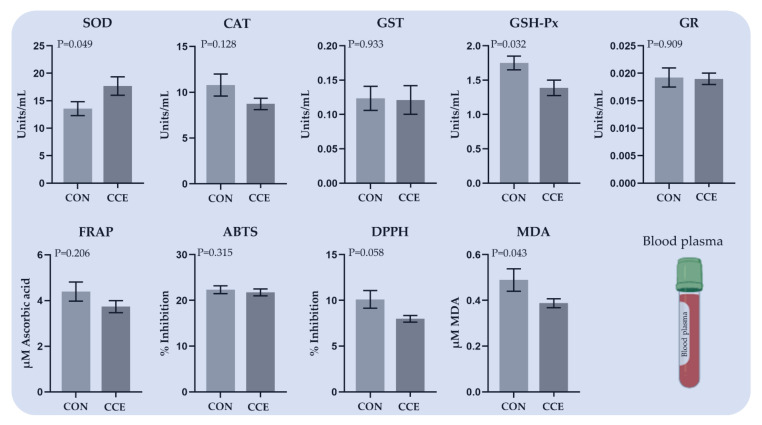
The mean ± SEM of total antioxidant capacity, oxidative stress indicators, and enzyme activities (Units/mL), in the blood plasma of broilers fed the two experimental diets (Control; CON, and carotenoid extract from *Citrus reticulata* included in soluble starch; CCE) at 42 days. *SOD: Superoxide Dismutase, CAT: Catalase, GST: Glutathione Transferase, GSH-Px: Glutathione Peroxidase, GR: Glutathione Reductase, FRAP: Ferric Reducing Ability of Plasma*, *ABTS: 2,2′-azinobis-(3-ethylbenzothiazoline-6-sulfonate), DPPH: 2,2′-diphenyl-1-picrylhydrazyl radical, and MDA: Malondialdehyde*.

The effects of feeding *Citrus reticulata* carotenoid extract on the antioxidant enzymes activities, total antioxidant capacity, and oxidative status in the blood plasma of broilers are presented in Figure 5. SOD activity was increased (*p* = 0.049) in the blood plasma of the CCE-fed broilers (Figure 5). The activity of GSH-Px in the blood plasma of the CEO-fed broilers was significantly decreased (*p* = 0.032) compared to the CON group (Figure 5). The total antioxidant capacity of blood plasma estimated by the DPPH method tended to decrease in the CCE- fed broilers (*p* = 0.058), while the lipid peroxidation indicator (MDA) was also substantially decreased (*p* = 0.043).

### 3.6. Transcriptional Profiling of Immune-Related Genes

#### 3.6.1. Relative Transcript Levels of Genes Regulating the Immune System in Liver

**Figure 6:**
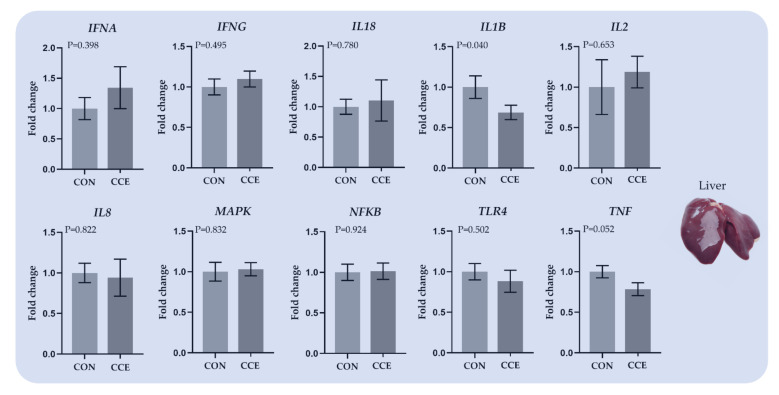
The mean and standard error of means (SEM) of relative transcript levels as fold changes of several genes involved in the immune system in the liver of broilers fed the two experimental diets (Control; CON and carotenoid extract from *Citrus reticulata* included in soluble starch; CCE) at 42 days. *INFA: Translation initiation factor IF-1, INFG: Interferon-gamma, IL18: Interleukin 18, IL1B: Interleukin 1 Beta, IL2: Interleukin 2, IL8: C-X-C Motif Chemokine Ligand 8, MAPK: Mitogen-activated protein kinase, NFKB: Nuclear factor-kappa B, TLR4: Toll-like receptors 4, and TNF: Tumor necrosis factor*.

The effects of feeding *Citrus reticulata* carotenoid extract on the relative expression of genes involved in the immune system in the liver of broilers are presented in Figure 6. The relative transcript levels of the proinflammatory cytokines *IL1B* and *TNF* were significantly downregulated (*p* = 0.040 and *p* = 0.052 respectively) in the liver of the CCE-fed broilers (Figure 6).

#### 3.6.2. Relative Transcript Levels of Genes Regulating the Immune System in the Spleen

**Figure 7:**
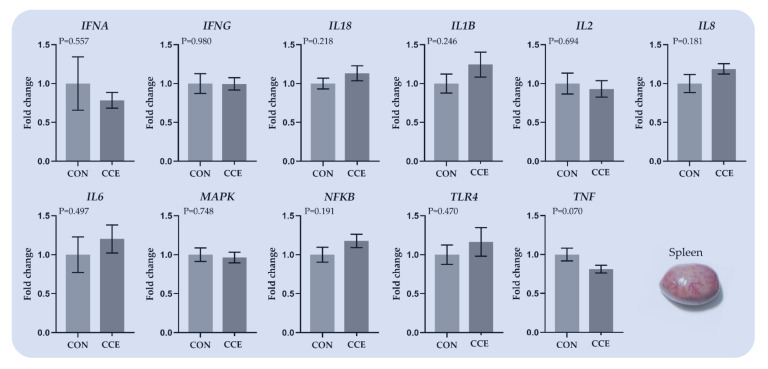
The mean and standard error of means (SEM) of relative transcript levels as fold changes of several genes involved in the immune system in the spleen of broilers fed the two experimental diets (Control; CON and carotenoid extract from *Citrus reticulata* included in soluble starch; CCE) at 42 days. *INFA: Translation initiation factor IF-1, INFG: Interferon-gamma, IL18: Interleukin 18, IL1B: Interleukin 1 Beta, IL2: Interleukin 2, IL8: C-X-C Motif Chemokine Ligand 8, IL6: Interleukin 6, MAPK: Mitogen-activated protein kinase, NFKB: Nuclear factor-kappa B, TLR4: Toll-like receptors 4, and TNF: Tumor necrosis factor*.

The effects of feeding *Citrus reticulata* carotenoid extract on the relative expression of genes involved in the immune system in the spleen of broilers are presented in Figure 6. The relative transcript levels of the proinflammatory cytokine *TNF* tended to downregulate (*p* = 0.070) in the spleen of the CCE-fed broilers (Figure 7).

#### 3.6.3. Relative Transcript Levels of Genes Regulating the Immune System in the Bursa of Fabricius

**Figure 8:**
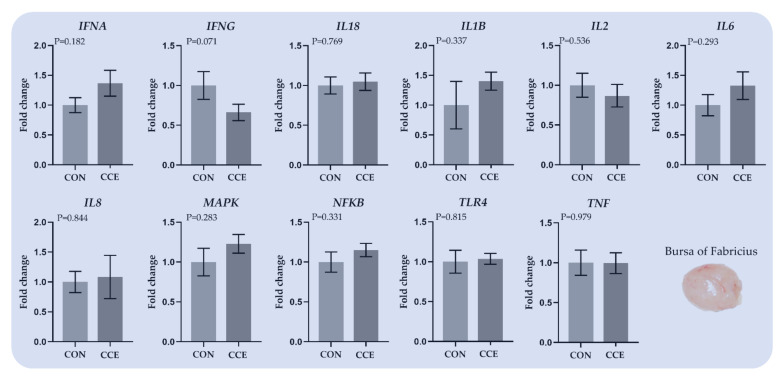
The mean and standard error of means (SEM) of relative transcript levels as fold changes of several genes involved in the immune system in the bursa of Fabricius of broilers fed the two experimental diets (Control; CON and carotenoid extract from *Citrus reticulata* included in soluble starch; CCE) at 42 days. *INFA: Translation initiation factor IF-1, INFG: Interferon-gamma, IL18: Interleukin 18, IL1B: Interleukin 1 Beta, IL2: Interleukin 2, IL8: C-X-C Motif Chemokine Ligand 8, IL6: Interleukin 6, MAPK: Mitogen-activated protein kinase, NFKB: Nuclear factor-kappa B, TLR4: Toll-like receptors 4, and TNF: Tumor necrosis factor*.

The effects of feeding *Citrus reticulata* carotenoid extract on the relative expression of genes involved in the immune system in the bursa of Fabricius are presented in Figure 6. The relative transcript levels of *INFG* tended to downregulate (*p* = 0.071) in the bursa of Fabricius of CCE compared to the CON-fed broilers (Figure 8).

## 4. Discussion

### 4.1. b-Cryptoxanthin: An Antioxidant Ally from Citrus reticulata

Β-cryptoxanthin was the prevailing carotenoid in *Citrus reticulata* extract. In agreement with our findings, Alquézar et al. [33], nominated b-cryptoxanthin as the dominant carotenoid in eight mandarin hybrids. Although *Citrus reticulata* (mandarin) and *sinensis* (orange) belong to the same genus, their carotenoids profile is quite diverse. More specifically, the dominant carotenoid in orange peel and pulp counting up to 80%, is the violaxanthin [33]. Interestingly, b-cryptoxanthin poses approximately seven-fold higher bioavailability compared to α- and β-carotene [34].

Hence, *Citrus reticulata* extract may exert beneficial outcomes in both human and animal nutrition in lower ingestion levels compared with other carotenoid-rich sources. CCE feed additive efficiently suppressed the relative viability of selected bacterial strains using the MTT assay. *Salmonella, Staphylococcus,* and *Escherichia* species are among the most commonly identified pathogenic bacteria in poultry [35,36]. 

Their presence results in severe antibiotic dependence in the poultry sector contributing to antimicrobial resistance (AMR). Although *Klebsiella* species are well-known as an opportunistic pathogen for broilers, multi-antibiotic resistant species threaten human health through meat and egg consumption [37]. The promising antimicrobial properties of *Citrus reticulata* extract portray a sustainable strategy to counteract infectious bacterial diseases and restrict the AMR outbreak. In the study of Karpinski and Adamczak [38], fucoxanthin an algae-derivate carotenoid effectively inhibited both Gram-positive and negative bacteria strains. 

More specifically, fucoxanthin had a significantly stronger impact on Gram-positive compared with on Gram-negative bacteria, while *E. coli* demonstrated the greatest inhibition compared to other Gram-negative strains. Generally, fucoxanthin appears to be a well-documented carotenoid toward bacterial growth, and its antimicrobial potential is quite limited against Gram-negative strains [39]. Thus, it is plausible to assume that the inhibitory properties of *Citrus reticulata* extract in both Gram-positive and -negative strains may lie on b-cryptoxanthin or to the synergistic action of different compounds present in the extract.

### 4.2. Although Broilers Performance and Carcass Traits Were Not Substantially Affected, Meat Oxidative Stability Was Slightly Improved

Dietary antioxidants constitute a promising strategy to inhibit an oxidative cascade driven by polyunsaturated fatty acids (PUFA) due to their high propensity to oxidation. The antioxidant capacity of *Citrus reticulata* extract may prevent PUFA oxidation prior to their deposition to tissues [40]. Considering this hypothesis, it is plausible to assume that the tendency for higher PUFA content in breast meat of the CCE-fed broilers was attributed to *Citrus reticulata* extract antioxidant properties. The decrease in the MDA concentration in the breast meat of the CCE- fed broilers supports the hypothesis regarding the antioxidative properties of *Citrus reticulata* extract. 

Although there is controversial evidence regarding the bioavailability of carotenoids in human and animal models [41], the improvement of meat oxidative stability through carotenoids dietary inclusion portrays a well-documented scenario [6]. In agreement with our study, the dietary supplementation with commercial rich in astaxanthin product decreased the TBARS content in broilers muscles kept in both thermoneutral and high ambient temperature conditions [42].

Considering trials using Citrus by-products, the dietary supplementation with either 2% orange or grapefruit peels decreased the TBARS concentration in broilers thigh meat after 7 days at 4 °C [43]. The improved oxidative stability of broilers meat could lie on either the deposition of antioxidant compounds in tissues or on the overall amelioration of the organism’s antioxidants systems. Although the cooking loss rate was higher in the CCE-fed broilers, the tendency for a higher carcass yield that was observed in this group could curve the aforementioned disadvantage.

### 4.3. Citrus Extract Modified Liver and Blood Oxidative Balance

Amongst the parameters investigated in broilers’ blood, aspartate aminotransferase (AST) was significantly decreased in the CCE-fed broilers. AST reflects the condition of liver function [44]. Overlooking the cytoprotective properties of carotenoids against liver oxidative damage by restraining or delaying the oxidation and suppressing ROS production, they are also considered precursors of retinol (vit A), which is involved in hepatic stellate cells rejuvenation, thereby, preventing the progression of fibrosis to hepatocellular carcinoma in humans [45]. 

In agreement with our findings, a combination of curcumin and lutein at 150 mg/kg in broiler diets reduced plasma ALT and AST levels in coccidiosis-induced chickens [46]. Although it appears that there is a strong linkage between AST and total cholesterol [46], our data did not confirm this scenario. The elevated cholesterol content in the carotenoids-fed birds is still questionable. Recent evidence suggests that supplementation with β-Carotene in human and mice trials could result in an upsurge of serum cholesterol in β-carotene oxygenase 1 (*BCO1*) deficient individuals [47]. However, further research should be applied on the topic to clarify this open question.

Regarding the antioxidant mechanisms, although *SOD* relative transcript level remained unaffected in the liver of the CEE fed broilers, its activity was significantly increased in the blood plasma indicating an efficient superoxide anion neutralization. SOD appears to be the first line of the antioxidant system by neutralizing the most unstable reactive oxygen species (ROS) superoxide anion (O_2_^•−^) to a less harmful radical—hydrogen peroxide (H_2_O_2_) [48]. 

This aspect of *Citrus reticulata* extract inclusion in broiler diets highlights the beneficial potential of its bioactive compounds since SOD activity is linked with the prevention of tissue damage caused by the pro-inflammatory response and DNA degradation and plenty of pathological conditions driven by lipid peroxidation and oxidative imbalances [49]. The close linkage between carotenoid consumption and SOD activity is further confirmed since it has been also found that plasma levels of lutein, lycopene, zeaxanthin, and alpha- and beta-carotene were substantially correlated with SOD activity in humans [50]. 

On the other hand, our study reveals insights that the formed hydrogen peroxide was unable to be effectively neutralized since the activity of the involved enzymes (CAT and GSH-Px) was suppressed. More specifically, *CAT*-relative transcript levels were substantially downregulated in the liver of the CCE-fed broilers following the same trends in the blood plasma. Even though *GPX1* and *GPX2* were not affected in the liver of the CCE-fed broilers, the activity of GSH-Px was decreased in the blood plasma. Various biological mechanisms could be attributed to CAT and GSH-Px inhibition in the *Citrus reticulata* extract-fed broilers. 

Primarily, the counterbalancing of O_2_^•−^ by SOD in blood and, subsequently, the production of H_2_O_2_, the main substrate of both CAT and GSH-Px, may result in inhibitory feedback. More specifically, it was previously found that a high concentration of H_2_O_2_ inhibits the activity of GSH-Px [51], while the hydroxyl radical formed by a Fenton reaction using H_2_O_2_ as a substrate inhibits CAT activity as well [52]. Secondly, it has also been found that flavonoids contained in *Citrus* essential oils, such as hesperidin, hesperetin, naringin, naringenin, diosmin, quercetin, rutin, nobiletin, tangeretin [53], could inhibit CAT and GSH-Px activity [54,55].

In agreement with our results, the dietary supplementation with spinach products (rich in both carotenoids and flavonoids) decreased human blood CAT activity compared with the control diet and a treated with a carotenoid blend (beta-carotene, lutein, and zeaxanthin) group [56]. Thus, Castenmiller et al. [56] attributed this downregulation to flavonoid substances of spinach.

However, it is of high interest the understanding of inhibitory mechanisms of specific flavonoids in solely GSH-Px activity, while the other glutathione-dependent enzymes (GR and GST) were not impaired. Regarding the lower CAT and GSH-Px activities, we could also speculate that non-enzymatic antioxidants compounds contained in *Citrus reticulata* extract were capable of neutralizing ROS, such as H_2_O_2_, thus, preserving the organism’s oxidative balance. Considering the above issues, it may be a future challenge to combine *Citrus* extracts with other bioactive herbal derivatives aiming to improve the second stage of the enzymatic antioxidative process; this of H_2_O_2_ neutralization.

Although the formation of hydroxyl radicals through hydroxyl peroxides are naturally capable to oxidize both feed and cell phospholipid PUFAs, the concentration of the principal lipid peroxidation biomarker MDA was significantly decreased. The activation of the SOD enzyme in the blood may be a substantial factor for MDA suppression. In accordance with our findings, several studies have reported the inhibitory properties of β-carotene, lycopene, lutein, and β-cryptoxanthin against lipid peroxidation in rat livers [45]. A potential mechanism that underlies the well-documented prevention action of carotenoids towards lipids peroxidation may be attributed to their strictly polar nature which binds them to phospholipids.

Numerous enzymes and cellular processes produce reactive oxygen species (ROS), as by-products of their catalytic function or from a dysfunctional variant of the enzyme. NADPH oxidases (NOX family) are the only enzymes whose principal role is to generate superoxide and consequently other ROS [57]. In our study, the relative transcript level of *NOX2* was decreased in the liver of the CCE-fed birds, indicating a lower production of ROS. Although there is adequate information regarding the inhibitory effect of polyphenolic compounds towards NOX activity [58], there is a knowledge gap concerning the impact of carotenoids.

Interestingly, NOX2 catalytic enzymes have been associated with pain signaling in the brain centers, and their regulation may conceal a pivotal perspective for productive animals’ welfare. Nevertheless, the downregulation of *NOX2* in the liver of the CCE-fed birds appears to be a promising perspective of *Citrus* by-products’ valorization in animal feeds.

### 4.4. The Pro-Inflammatory Mediators Were Downregulated at Transcriptional Level

Various factors, such as disease, heat stress, and environmental conditions can threaten broilers’ immune-oxidative balance. Even though the principal regulatory factors of proinflammatory response, such as *TLR4* and *NFkB,* were not affected, the pro-inflammatory cytokines *IL1B* and *TNF* were downregulated in the livers of the CCE-fed broilers while *TNF* was also suppressed in the spleens. Additionally, *IFNG* was downregulated in the bursa of Fabricius of the CCE-fed broilers as well. IL-1β and TNF-α are pro-inflammatory cytokines that regulate the inflammatory reactions and stimulate T cells and macrophages [59].

Interferon-γ (IFN-γ) is another pro-inflammatory cytokine, which also activates the macrophages and is produced by Th1 cells [59]. Astaxanthin revealed an anti-inflammatory effect in LPS-treated mice via the inhibition of the pro-inflammatory cytokines, such as TNF-α and IL-1β [60]. Other xanthophylls could also downregulate the gene expression levels of pro-inflammatory IL-1β, interleukin-6 (IL-6), and IFN-γ in hens [61]. Furthermore, both beta-carotene and lycopene inhibited the *TNF* gene expression in RAW264.7 cells [62].

Transcriptome analysis reveals that the most important mechanism for maintaining immune homeostasis with dietary carotenoid supplements was their antioxidant properties and especially their regulatory effects on NADPH oxidases [63]. However, it remains an open question whether the NADPH oxidases downregulated the pro-inflammatory cytokines or the suppression of *IL1B* and *TNF* transcript levels downregulated the *NOX2.* Elucidating this scientific gap, in the study of Yang et al. [64], the inhibition of NADPH oxidase suppressed the production of pro-inflammatory cytokines IL-1β and IFNγ. Additionally, evidence suggests that IL-1β could upregulate the production of NOX in cultured smooth muscle cells from the human coronary artery [65].

These preliminary data on the transcriptional level praise the immunomodulatory properties of *Citrus reticulata* extracts in poultry. The latter possesses great importance for poultry’s health and welfare since non-infectious stimuli, such as environmental conditions, stocking density, and dietary antigens, for instance beta-conglycinin, present in soybean meal, as well as cereal gluten prolamins, can lead to low-grade chronic inflammation [66,67].

Even though our study provided new insights regarding the beneficial properties of carotenoids contained in *Citrus reticulata* in broilers’ immune-oxidative status and their in vitro antimicrobial potential, there are still plenty of open questions. Notably, we should evaluate if the *Citrus reticulata* extract is capable of partially substituting the synthetic antioxidants in the poultry sector under challenging conditions. The effect of CCE extract should be also evaluated in both intestinal microbiome structure and immune response aiming to holistically assess its potential as a bioactive feed additive.

## 5. Conclusions

The utilization of juice industry by-products appears to be a sustainable bioprocess, including compounds with both antimicrobial and antioxidative potential. Our results suggest that broilers’ immune-oxidative status was slightly improved while insight for beneficial outcomes in products quality was unveiled. The tight linkage between the antioxidant mechanisms and immune regulation under the influence of carotenoid blends administration should be further explored, while the antimicrobial potential of *Citrus reticulata* extract needs to be validated in vivo under challenging field conditions.

## Figures and Tables

**Table 1:** Calibration curve equations, R2, and retention times of each analyte.

Carotenoids	Equation	R^2^	Retention Time (min)
Fucoxanthin	Y = −5095.71 + 64,509.9X	0.9987	2.73
Astaxanthin	Y = −8606.49 + 44,409.2X	0.9996	4.67
Lutein	Y = −8753.93 + 302,049X	0.9996	5.66
Zeaxanthin	Y = −44,000.3 + 629,033X	0.9995	6.40
Canthaxanthin	Y = −5054.97 + 13,102.6X	1.0000	8.45
β-Cryptoxanthin	Y = −4777.46 + 33,248.5X	0.9997	13.90
α-Carotene	Y = −182,698 + 9.24063e + 0.006X	0.9997	22.59
β-Carotene	Y = 7951.8 + 2.9564e + 0.006X	0.9993	25.64

**Table 2:** Composition (%) and chemical analysis (%) of the starting (0–10 d), growing (11–24 d), and finishing (25–42 d) phase of the control (CON) and *Citrus* carotenoid extract (CCE) diets.

Ingredients	Dietary Treatment					
	CON	CCE	CON	CCE	CON	CE
	Starter Period (Day 1–10)		Grower Period (Day 11–24)		Finisher Period (Day 25–42)	
Composition %						
Maize	50.52	50.42	53.90	53.80	59.35	59.25
Soyabean meal	40.89	40.89	37.09	37.09	31.49	31.49
Soybean oil	4.03	4.03	4.97	4.97	5.41	5.41
Vitamin and mineral premix ^1^	0.2	0.2	0.2	0.2	0.2	0.2
Limestone	1.61	1.61	1.47	1.47	1.34	1.34
NaCl	0.40	0.40	0.4	0.4	0.40	0.40
Monocalcium phosphate	1.43	1.43	1.22	1.22	1.09	1.09
Methionine	0.41	0.41	0.36	0.36	0.34	0.34
Lysine	0.27	0.27	0.20	0.20	0.21	0.21
Threonine	0.15	0.15	0.11	0.11	0.08	0.08
Choline	0.09	0.09	0.08	0.08	0.09	0.09
Citrus carotenoid extract included in starch	-	0.1	-	0.1	-	0.1
Chemical analysis %						
Dry matter %	91.5	90.5	91.4	89.9	90.0	91.1
Ash %	6.7	6.6	6.7	6.6	6.7	6.6
Crude protein %	24	23.9	21.9	21.4	19.8	19.8
Ether exctract %	5.7	5.6	6.6	6.4	7.3	7
Crude fiber %	2.2	2.4	2.3	2.4	2.3	2.5
ME (MJ/kg)	12.5	12.5	13	13	13.4	13.4
Sodium %	0.16	0.16	0.16	0.16	0.16	0.16
Calcium %	0.96	0.96	0.87	0.87	0.79	0.79
Phosphorus %	0.48	0.48	0.43	0.43	0.4	0.4
Lysine %	1.44	1.44	1.29	1.29	1.16	1.16
Methionine and cysteine	1.08	1.08	0.99	0.99	0.91	0.91
Threonine %	0.97	0.97	0.88	0.88	0.78	0.78

^1^ Premix supplied per kg of diet: 13,000 IU vitamin A (retinyl acetate), 3500 IU vitamin D3 (cholecalciferol), 70 mg vitamin E (DL-α-tocopheryl acetate), 7 mg vitamin K3, 8.5 mg thiamin, 8 mg riboflavin, 5 mg pyridoxine, 0.020 mg vitamin B12, 50 mg nicotinic acid, 15 mg pantothenic acid, 1.5 mg folic acid, 0.15 mg biotin, 1 mg iodine, 50 mg iron, 75 mg manganese, 15 mg copper, 0.3 mg selenium, and 75 mg zinc.

**Table 3:** Sequences and relative positions of primers for target genes used in real-time qPCR.

Gene	Sequence	Amplicon bp	Accession No. *	References
*GAPDH*	F: 5′- GCTGGCATTGCACTGAATGAC -3′	113	NM_204305.1	[4]
	R: 5′- CACTCCTTGGATGCCATGT -3′			
*ACTB*	F: 5′- AGCGAACGCCCCCAAAGTTCT -3′	139	NM_205518.1	[4]
	F: 5′- AGCTGGGCTGTTGCCTTCACA -3′			
*CAT*	R: 5′- TGGCGGTAGGAGTCTGGTCT -3′	112	NM_001031215.1	[4]
	R: 5′- GTCCCGTCCGTCAGCCATTT -3′			
*GPX1*	F: 5′- AACCAATTCGGGCACCAG -3′	122	NM_001277853.2	[4]
	R: 5′- CCGTTCACCTCGCACTTCTC -3′			
*GPX2*	F: 5′- GAGCCCAACTTCACCCTGTT -3′	75	NM_001277854.2	[4]
	R: 5′- CTTCAGGTAGGCGAAGACGG -3′			
*SOD1 (CuZn)*	F: 5′- CACTGCATCATTGGCCGTACCA -3′	224	NM_205064.1	[4]
	R: 5′- GCTTGCACACGGAAGAGCAAGT -3′			
*GSTA2*	F: 5′- GCCTGACTTCAGTCCTTGGT -3′	138	XM_015284825.3	[4]
	R: 5′- CCACCGAATTGACTCCATCT -3′			
*NOS2*	F: 5′- AAAGAAAGGGATCAAAGGTGGT -3′	296	NM_204961.1	[4]
	R: 5′- CAAGCATCCTCTTCAAAGTCTG -3′			
*NOX1*	F: 5′- TCATCACTCTGGCGCTCATC -3′	171	XM_040698828.1	[4]
	R: 5′- CCTTCATGCTCTCCTCCGTC -3′			
*NOX2*	F: 5′- TGGTGCGGTTTTGGAGATCA -3′	145	XM_040698636.1	[4]
	R: 5′- GACACTGCTGGGCATTTGAC -3′			
*NOX3*	F: 5′- TTGGAATGGGAGAAGGCCAC-3′	92	XM_040667279.1	[4]
	R: 5′-AGCACCACAGGACTCACAAC-3′			
*TLR4*	R: 5′- ACCCGAACTGCAGTTTCTGGAT -3′	120	NM_001030693.1	[27]
	R: 5′- AGGTGCTGGAGTGAATTGGC -3′			
*MAPK*	F: 5′- GAACGTGCGCTTCATCTACG -3′	137	XM_040649449.1	[27]
	R: 5′- CCACGGGCTTAAACGCTTTC -3′			
*NFKB*	F: 5′- GAAGGAATCGTACCGGGAACA -3′	131	NM_205134 131	[27]
	R: 5′- CTCAGAGGGCCTTGTGACAGTAA -3′			
*TNF*	F: 5′- CCCCTACCCTGTCCCACAA -3′	67	NM204267	[27]
	R: 5′- TGAGTACTGCGGAGGGTTCAT -3′			
*INFA*	F: 5′- ACTTCAGCTGCCTCCACACCTT -3′	92	AM049251.1	[27]
	R: 5′- CAGGAACCAGGCACGAGCTT -3′			
*INFG*	F: 5′- AACAACCTTCCTGATGGCGTGA -3′	89	NM_205149.1	[27]
	R: 5′- GCTTTGCGCTGGATTCTCAAGT -3′			
*IL1B*	F: 5′- TGCTTCGTGCTGGAGTCACCC -3′	98	XM_015297469.1	[27]
	R: 5′- GGCCGGTACAGCGCAATGTT -3′			
*IL2*	F: 5′- CGTAAGTGGATGGTTTTCCTCT -3′	161	NM204153	[27]
	R: 5′- GGCTAAAGCTCACCTGGGTC -3′			
*IL6*	F: 5′- AGCGAAAAGCAGAACGTCGAGTC -3′	107	XM_015281283.2	[27]
	R: 5′- GCCGAGTCTGGGATGACCACTTC -3′			
*IL8*	F: 5′- CTGGCCCTCCTCCTGGTT-3′	105	HM179639	[27]
	R: 5′- GCAGCTCATTCCCCATCTTTAC -3′			
*IL18*	F: 5′- GTTGTTCGATTTAGGGAAGGAG -3′	146	NM204608.1	[27]
	R: 5′- TCAAAGGCCAAGAACATTCC -3′			

* Ref Seq: NCBI Reference Sequence database.

**Table 4:** Carotenoid composition in the non-volatile fraction of mandarin (*Citrus reticulata*) CPEO.

Carotenoids	Before Saponification (mg/g Non-Volatile Fraction)	After Saponification (mg/g Non-Volatile Fraction)	Total Carotenoids (mg/g Non-Volatile Fraction)	Total Carotenoids mg/100 g Starch Feed Additive, CCE)
Fucoxanthin	n.d.	n.d.	n.d.	n.d.
Astaxanthin	tr	tr	tr	tr
Lutein	tr	0.033 ± 0.001	0.033 ± 0.001	0.082 ± 0.002
Zeaxanthin	tr	0.10 ± 0.01	0.10 ± 0.01	0.250 ± 0.002
Canthaxanthin	n.d.	n.d.	n.d.	n.d.
β-Cryptoxanthin	1.62 ± 0.04	8.7 ± 0.7	10.3 ± 0.7	22 ± 2
α-Carotene	0.011 ± 0.008	tr	0.011 ± 0.008	tr
β-Carotene	0.143 ± 0.009	0.132 ± 0.002	0.143 ± 0.009	0.36 ± 0.02

Values are means ± standard deviation, n.d., not detected, tr, traces.

**Table 5:** IC50 in mg CCE extract/mL of MTT assay.

Bacteria	Equation	R^2^	IC50 mg/mL
*Escherichia coli*	y = −0.0268x + 0.7314	0.9537	8.63
*Klebsiella oxytoca*	y = −0.0256x + 1.038	0.9591	21.02
*Salmonella typhimurium*	y = −0.0141x + 0.6859	0.9803	13.18
*Staphylococcus aureus*	y = −0.0661x + 0.7787	0.8957	4.22

**Table 6:** Broiler growth performance on starter, grower, finisher, and overall experimental period among the two dietary treatments (Control; CON and carotenoid extract from *Citrus reticulata* included in soluble starch; CCE).

	Dietary Treatment			
	CON	CCE	SEM	Significance
Initial BW (g)	44.08	44.09	0.54	0.980
Day 1–10				
BW (g)	276.9	288.2	13.74	0.441
AFI (g)	288.7	290.1	10.88	0.906
Day 11–24				
BW (g)	1229	1261	34.79	0.386
AFI (g)	1195	1215	29.28	0.525
Day 25–42				
BW (g)	2976	3056	100.50	0.456
AFI (g)	2722	2787	118.40	0.603
Day 1–42				
AFI (g)	4205	4292	148.40	0.582
FCR	1.43	1.41	<0.001	0.450
Mortality (%)	0	0	-	-
Carcass yield (%)	75.81	76.47	0.655	0.093
Spleen (% of BW)	0.094	0.083	0.011	0.241
Liver (% of BW)	1.59	1.54	0.048	0.467
Bursa of Fabricius (% of BW)	0.071	0.060	0.011	0.105

Final BW: final body weight; FI: feed intake; FCR: feed conversion ratio (g feed/g gain); and SEM: pooled standard error of means. The spleen, liver, and bursa of Fabricius are expressed as a percentage of final body weight (g/100 g body weight).

**Table 7:** Carcass quality based on selected parameters among the two dietary treatments (Control; CON and carotenoid extract from *Citrus reticulata* included in soluble starch; CCE).

	Dietary Treatment			
	CON	CCE	SEM	Significance
Color traits				
L*	55.87	57.56	1.714	0.342
a*	7.18	7.05	0.979	0.891
b*	18.50	18.49	1.434	0.995
Physical traits				
pH24	6.11	6.04	0.089	0.440
Cooking loss (%)	11.83	15.39	1.612	0.044
Shear force (100 N/mm^2^)	14.22	14.15	1.663	0.967

L* = lightness, a* = redness, b* = yellowness.

## Data Availability

All data are contained within the article.

## Supplementary Materials

The following supporting information can be downloaded at: https://www.mdpi.com/article/10.3390/antiox11010144/s1, Table S1: The mean individual fatty acids (FA) (% of total FA) in breast meat of chickens fed the CON and CEO diets at 42nd day.
