# Supplementary material for: Effect of a Carotenoid Extract from Citrus reticulata By-Products on the Immune-Oxidative Status of Broilers
Source: Antioxidants (Basel). 2022 Jan 10;11(1):144. doi: 10.3390/antiox11010144 (PMC8773417; doi:10.3390/antiox11010144)
Supplement: Supplementary file 1 [file antioxidants-11-00144-s001.zip › antioxidants-1503258-supplementary.pdf]

Table S1. The mean individual fatty acids (FA) (% of total FA) in breast meat of chickens fed the CON and CEO diets at 42nd day.

	Dietary treatment			
**Fatty acids**	**CON**	**CCE**	**SEM**	**Significance**
Myristic acid ($C_{14:0}$)	0.325	0.297	0.008	0.560
Pentadecanoic acid ($C_{15:0}$)	0.23	0.19	0.009	0.409
Palmitic acid ($C_{16:0}$)	17.12	16.74	0.85	0.394
Palmitoleic acid ($C_{16:1\ n\text{-}7}$)	1.18	1.27	0.27	0.522
Margaric acid ($C_{17:0}$)	0.12	0.11	0.05	0.704
Stearic acid ($C_{18:0}$)	8.16	7.91	0.72	0.498
$C_{18:1\ trans}$	0.02	0.047	0.07	0.499
Oleic acid ($C_{18:1\ cis\text{-}9}$)	23.93	23.23	1.428	0.346
cis-Vaccenic acid ($C_{18:1\ cis\text{-}11}$)	1.693	1.698	0.112	0.931
Linoleic acid ($C_{18:2\ n\text{-}6\ cis}$)	32.16	33.26	2.214	0.323
α- Linolenic acid ($C_{18:3\ n\text{-}3}$)	2.835	2.933	0.434	0.657
γ- Linolenic acid ($C_{18:3\ n\text{-}6}$)	0.20	0.245	0.098	0.363
Eicosadienoic acid ($C_{20:2\ n\text{-}6}$)	0.74	0.656	0.119	0.184
Eicosatrienoic acid ($C_{20:3\ n\text{-}6}$)	0.702	0.776	0.168	0.393
Arachidonic acid ($C_{20:4\ n\text{-}6}$)	9.04	9.13	2.034	0.942
Docosadienoic acid ($C_{22:2\ n\text{-}6}$)	0.164	0.170	0.074	0.872
Eicosapentanoic acid ($C_{22:5\ n\text{-}6}$)	0.791	0.822	0.201	0.770
Docosahexaenoic acid ($C_{22:6\ n\text{-}3}$)	0.532	0.456	0.185	0.459
Saturated fatty acids (SFA)	25.96	25.24	1.301	0.290
Unsaturated fatty acids (USFA)	73.99	74.69	1.346	0.313
SFA/ UNFA	0.351	0.338	0.024	0.296
Monounsaturated fatty acids (MUFA)	26.82	26.25	1.649	0.499
Polyunsaturated fatty acids (PUFA)	47.17	48.45	1.689	**0.096**
Atherogenic Index (AI)	0.25	0.24	0.017	0.321

Atherogenicity index (AI) was calculated according to the equation: (C12:0 + 4 × C14:0 + C16:0)/(PUFA + MUFA), as described by Mavrommatis et al. [1].

1. Mavrommatis, A.; Sotirakoglou, K.; Kamilaris, C.; Tsiplakou, E. Effects of Inclusion of Schizochytrium spp. and Forage-to-Concentrate Ratios on Goats' Milk Quality and Oxidative Status. *Foods* **2021**, *10*, 1322, doi:10.3390/foods10061322.
